# Childhood disability in Malawi: a population based assessment using the key informant method

**DOI:** 10.1186/s12887-017-0948-z

**Published:** 2017-11-28

**Authors:** Myroslava Tataryn, Sarah Polack, Linda Chokotho, Wakisa Mulwafu, Petros Kayange, Lena Morgon Banks, Christiane Noe, Chris Lavy, Hannah Kuper

**Affiliations:** 1 International Centre for Evidence in Disability, London School of Hygiene & Tropical Medicine, WC1E 7HT, London, UK; 2 Beit Cure International Hospital, Blantyre, Malawi; 3 Department of Surgery, College of Medicine, University of Malawi, Blantyre, Malawi; 4 Department of Surgery, Opthalmology Unit, College of Medicine, University of Malawi, Blantyre, Malawi; 5 CBM, Bensheim, Germany; 6 Nuffield Department of Orthopaedics Rheumatology and Musculoskeletal Science, Oxford University, Oxford, UK

**Keywords:** Children, Disability, Impairment, Key informant method, Malawi, Physical impairments, Sensory impairments, Intellectual impairments, Epilepsy, Service needs

## Abstract

**Background:**

Epidemiological data on childhood disability are lacking in Low and Middle Income countries (LMICs) such as Malawi, hampering effective service planning and advocacy. The Key Informant Method (KIM) is an innovative, cost-effective method for generating population data on the prevalence and causes of impairment in children. The aim of this study was to use the Key Informant Method to estimate the prevalence of moderate/severe, hearing, vision and physical impairments, intellectual impairments and epilepsy in children in two districts in Malawi and to estimate the associated need for rehabilitation and other services.

**Methods:**

Five hundred key informants (KIs) were trained to identify children in their communities who may have the impairment types included in this study. Identified children were invited to attend a screening camp where they underwent assessment by medical professionals for moderate/severe hearing, vision and physical impairments, intellectual impairments and epilepsy.

**Results:**

Approximately 15,000 children were identified by KIs as potentially having an impairment of whom 7220 (48%) attended a screening camp. The estimated prevalence of impairments/epilepsy was 17.3/1000 children (95% CI: 16.9–17.7). Physical impairment (39%) was the commonest impairment type followed by hearing impairment (27%), intellectual impairment (26%), epilepsy (22%) and vision impairment (4%). Approximately 2100 children per million population could benefit from physiotherapy and occupational therapy and 300 per million are in need of a wheelchair. An estimated 1800 children per million population have hearing impairment caused by conditions that could be prevented or treated through basic primary ear care. Corneal opacity was the leading cause of vision impairment. Only 50% of children with suspected epilepsy were receiving medication. The majority (73%) of children were attending school, but attendance varied by impairment type and was lowest among children with multiple impairments (38%).

**Conclusion:**

Using the KIM this study identified more than 2500 children with impairments in two districts of Malawi. As well as providing data on child disability, rehabilitation and referral service needs which can be used to plan and advocate for appropriate services and interventions, this method study also has an important capacity building and disability awareness raising component.

## Background

Recent estimates suggest that 5% of all children – 93 million children globally – are living with moderate or severe disabilities [1]. The vast majority (80%) of these children reside in (low and middle income countries) LMICs [1], defined by the World Bank according to their Gross National Income Per Capita as Low: <1005; *Lower middle*: 1006–3955; Upper Middle: $3956–12,235 [2]. There is increasing evidence that children with disabilities are more likely to come from poorer households, are substantially less likely to attend school and experience poorer health compared to their non-disabled peers [1, 3].

There is very little reliable data on the epidemiology of impairments in children and the disabling factors they experience particularly in LMICs. This is partly due to the lack of available comparable data collection tools and definitions of child disability [1]. Malawi is a low income country [2] and one of the poorest in the world [4]. It has ratified the United Nations Convention on the Rights of the Child [5] (CRT) and the United Nations Convention on the Rights of Persons with Disabilities (UNCRPD) [6]. These two conventions – CRT adopted by the UN General assembly in 1989 and the UNCRPD in 2006 - mutually reinforce each other in promoting the changes needed to ensure that children with disabilities are guaranteed their human rights on an equal basis with others [7]. The implementation of both conventions are monitored at the international level by Committees which assess the progress of Member States. However, reliable information on the prevalence and types of impairment in children and the service needs for this population in Malawi are lacking. The 2008 Malawi Housing and Population Census estimated the overall prevalence of disability to be 2.4% among children and 3.8% in the general population [8]. However, this survey did not use disability measurement tools designed for children and there was no verification of self-reported functional limitations by clinical examination which limits its use in planning health and rehabilitation services. These data are urgently needed to plan appropriate and accessible services and for evidence based advocacy for children with disabilities.

The Key Informant Method (KIM) is an innovative method for generating population level data on the prevalence and causes of impairments in children [9, 10]. The method provides an important alternative to population based surveys which can be time consuming and costly. KIM involves training volunteers (Key Informants, KIs) to identify children in their communities who may have disabling impairments. The children are invited to attend a screening camp where they are examined by relevant medical professionals and referred to appropriate services as available. As well providing data on child disability and service needs, the KIM approach engages with local communities and stakeholders and has an important capacity building and disability awareness raising role [11]. The KIM has been used to identify epilepsy [12], childhood blindness [9] and maternal mortality [13] in the community and was found to be a valid and low cost method to assess child disability in Bangladesh [9]. However, it has not previously been used in Africa where a large disability data gap remains.

The aim of this study was to use the Key Informant Method in two districts in Malawi to estimate the prevalence of moderate/severe hearing, vision and physical impairment, intellectual impairment and epilepsy in children.

## Methods

### Study setting and population

The study was undertaken in Thyolo and Ntcheu districts in the Southern and Central regions of Malawi respectively, during April to November, 2013. These rural districts were selected with consideration to availability and proximity of health and rehabilitation services including in Blantyre and the Community Based Rehabilitation Programme in Ntcheu district. According to the 2011 Malawi National Integrated Household Survey [14] these districts are comparable to, or slightly above, the average of all rural districts in their respective regions in terms of key socio-economic indicators. The Rural South of Malawi is generally considered poorer than Rural Central [14].

In each district four out of the eight Traditional Authorities were included: Mpando, Kwataine, Niolomole, Goodson Ganya (Ntcheu) and Phuka, Chimaliro, Byumbwe, Thomas (Thyolo). This gave an estimated total study population of 338,200 children aged less than 18 years according to the 2008 census data, updated to reflect population growth.

### Definition of disability

The UNCRPD defines disability as the “Long-term physical, mental, intellectual or sensory impairments which, in interaction with various barriers, may hinder [a person’s] full and effective participation in society on an equal basis with others” [6]. The World Health Organisation International Classification of Functioning, Disability and Health (ICF) is a biopsychosocial model of disability that incorporates health conditions and functional impairments, activity limitations and participation restrictions as well as environmental barriers [15]. This study focuses on the impairment component of disability which is defined by the ICF as a ‘loss or abnormality in body structure or physiological function’. In LMICs, where access to medical treatment, rehabilitation and access to education and other services is limited, people with impairments are often disabled [16] but we did not specifically measure activities, participation or environmental components of disability in this study.

Specifically, this study measured moderate or severe hearing, vision and physical impairments, intellectual impairment and epilepsy. Epilepsy was also included because it is a health condition that can be potentially disabling: previous research has shown an association both between epilepsy and lower health-related quality of life, and between accidents during seizures and long term physical impairment. We did not assess mental disorders, such as depression, because of the lack of available tools for assessing this among children in low-income settings.

### Key Informants

Villages/communities in the study districts have volunteers, supervised by Health Surveillance Assistants, who regularly assist with public health campaigns and community mobilization. Five hundred KIs (250 per district) KIs were identified from among these existing groups of volunteers. The KIs were selected by Area Coordinators (Health Surveillance Assistants responsible for volunteers in a given area) following discussions with the study team and the District Health Officers and District Environmental Health Officers.

The volunteers and Area Coordinators attended a one-day training workshop which included: disability sensitization, identification of the specific impairments included in the study, methods for case finding, procedures of the screening camps. These training workshops, each of which included approximately 20 KIs, were coordinated and led by the Malawian Project Co-ordinator. After training KIs returned to their communities where, during a 3–6 week period, they identified children suspected to have one or more of the impairments/epilepsy included in the study. The Area Coordinators were responsible for supporting and monitoring the work of the KIs during this period. Identified children were listed in a register and invited together with a guardian to attend the nearest screening camp. The locations for the screening camps were determined in consultation with the Area Coordinators for each traditional authority. Area Coordinators were provided with fuel, mobile phone costs and an honorarium for the days spent on the study. KIs were paid a per diem and transport reimbursement for the training and day(s) spent at the screening camps.

### Disability assessment

Thirty-three screening camps were held throughout the study areas: 15 camps in Ntcheu and 18 in Thyolo. Children were screened for the eligible impairments by a team of professionals which comprised an orthopedic clinical officer, Ear Nose Throat (ENT) clinical officer, audiologist, ophthalmic clinical officer, nurses, social worker, rehabilitation technician. Up to three professionals of each clinical type were trained in the study protocol enabling them to rotate between attending the camps and clinical duties. Minimizing the number of clinicians was considered important for limiting the variability between examiners. The team underwent a one-day training on the organization of the screening camp and clinical examination protocols. Field supervisors (Malawi project coordinator and researcher from the London School of Hygiene & Tropical Medicine) attended the screening camps to monitor the quality of data collection and ensure consistency in protocol.

Assessment at the camps was conducted in three stages. Firstly, the caregiver was asked a set of six screening questions used to identify children at risk of vision, hearing, physical and intellectual impairment and epilepsy. Secondly, children with reported problems in one or more domains were invited to undergo the relevant clinical examinations as follows:

- Does your child have problems seeing? (If yes, vision assessment)
- Does your child have problems hearing? (If yes, hearing assessment)
- Does your child have a problem with their body that makes it hard for them to do daily activities like feeding or washing? (If yes, orthopaedic assessment)
- Does your child have problems walking? (If yes, orthopaedic assessment)
- Does your child have problems with learning/understanding (If yes, intellectual assessment)
- Does your child have problems talking? (If yes, hearing and intellectual assessment)
- Does your child have fits/convulsions (If yes, epilepsy assessment)

The clinical assessment for each impairment and epilepsy were conducted by an appropriate medical professional using standardised protocols and definitions as shown in Table 1. The protocols for assessing vision [17, 18], hearing [19] and physical impairment [20] were taken from standardized survey tools developed for and previously tested in LMICs. In the absence of standardized tools suitable for LMIC Intellectual Impairment was assessed using questions developed for this study in consultation with a local Occupational Therapist. Intellectual impairment was only assessed for children aged ≥2 years. Thirdly, any children confirmed to have vision, hearing or physical impairments were then examined by the ophthalmic clinical officer, ENT clinical officer or orthopedic clinical officer, respectively, to determine the cause of impairment.

**Table 1:** Clinical assessment method and definitions of impairment

Impairment/health condition	Screen/Exam	Ages (year)	Assessment	Case definition
Moderate/severe vision impairment	SCREEN	0–2	Fix and follow	Unable to fix and follow
		3–4	Counting fingers (child copies number of fingers shown at 6 m both eyes together)	Unable to count finger at 6 m (approx. Equivalent to Visual Acuity (VA) < 6/60).
		≥ 5	Visual acuity test using tumbling E-chart	Presenting VA <6/60 in better eye.
	EXAM		Eye exam with direct ophthalmoscope and retinoscope by ophthalmic clinical officer	
Moderate/severe hearing impairment	SCREEN	6 m-4	Otoacoustic Emissions (OAE) tests.	Fails OAE both ears.
		≥ 5	Ages ≥ 5 years: Pure Tone Audiometry (PTA)^a^	> 35 dBHL in both ears^a^
	EXAM		Ear exam by ENT clinical officer using an otoscope	
Moderate/severe physical impairment			Standardised observation of activities (ability to hold and change position, mobility, and hand function) and physical examination by orthopaedic clinical officer to determine severity and cause	Moderate/severe physical impairment lasting more than one month/from birth) affecting functioning based on observation of activities and physical examination.
Epilepsy			Eight screening questions about type and frequency of epilepsy episodes in the past year	Paediatric clinical officer/nurse confirmed epilepsy based on responses to screening questions.
	SCREEN	0–2		Paediatric clinical officer/Nurse judgement
Intellectual impairment		≥ 5	12 age-relevant questions on behaviour, communication, comprehension, concentration, relationships and learning ^b^	Scores positive on at least 3 questions and/or presence of Down’s Syndrome, microcephaly or hydrocephaly.

^a^It was not possible to undertake Pure Tone Audiometry 40% children aged ≥ 5 years due to noisy environments or communication difficulties. Children who could not undergo PTA were classified according to the Ottaoacoustic Emmissions test results. ^b^These questions were developed in consultation with local occupational therapist

### Covariates

We also collected socio-demographic data including the age, sex and school attendance of the child, caregiver literacy and family income.

### Sub-study of children who did not attend screening camps

Approximately half of the children listed by the KIs as potentially having an impairment/epilepsy did not attend a screening camp. The reason for this was unclear, but has important implications for future application of this methodology. We therefore conducted a sub-study to explore reasons for non-attendance. The caregivers of 295 children randomly selected from those who had not attended a camp were interviewed using a structured questionnaire about their reasons for not attending. It was not feasible, within the resources available, to conduct clinical examinations of children in the sub-study (as done in the camps) to assess prevalence of impairments among non-attenders. Therefore we assessed reported functioning of the child using the Washington Group short question set which asks about difficulty with six domains: hearing, seeing, walking, remembering or concentrating, self-care and being understood [21, 22]. Each domain has four possible response options ranging from “no difficulty” to “cannot do at all”. The Washington Group tool is widely used internationally in census and surveys to identify people at risk of disability [21].

### Data analysis

The denominator used to calculate the prevalence of impairments is the total number of children (338,235) living in the eight traditional areas in the two study districts. This figure is taken from the 2008 census, updated to reflect population growth. Not all the children identified by KIs attended the screening camps. Therefore, in order to estimate the total prevalence across the study areas, we made the assumption that the following were the same among children who did and did not attend the camps: a) the total proportion of children with any impairment/epilepsy and b) the distribution of impairments/epilepsy. We undertook sensitivity analysis of the prevalence of impairments/epilepsy (overall and for the individual impairment types) by varying the assumed proportion of non-attending children with impairments/epilepsy to ±10% of the proportion observed among the attenders. We estimated the number of children with impairments per million population as this is a useful figure for advocacy and planning. This was calculated using a method applied in previous published studies [18, 20] as follows: 1) the proportion of the population in the study area that were <18 years was calculated using 2008 census data and 2) the impairment prevalence estimate was multiplied by the proportion of children <18 years and then from per 1000 to per million (× 1000) to reach an estimate per 1 million total population. Prevalence of impairments within each district were also estimated. Logistic regression analysis was used to compare school attendance by impairment type.

### Ethical considerations

Ethical approval was obtained from the College of Medicine Research Ethics Committee, Malawi and the London School of Hygiene and Tropical Medicine. The study purpose and procedures were explained to the child and the accompanying parent/caregiver and signed/thumb-printed consent was obtained from the parents/caregiver of all participating children. Prior to the survey, we conducted a comprehensive mapping of the available referral services (e.g. Community Based Rehabilitation (CBR) programmes, ophthalmic, ENT and orthopaedic services) through discussions with local stakeholders and service providers. This is essential to ensure there are services available that are able to accommodate additional demand generated by the study. Children and their guardians identified as having an impairment/epilepsy were referred to onward services as appropriate.

## Results

### Study population

Data from 380 out of the 500 (76%) key informant registers that were available at the end of the project showed that each KI listed an average of 30 children as potentially having an impairment/epilepsy. Based on this, we estimated that a total of 15,000 children were referred by KIs to the screening camps. Of the estimated 15,000 children identified by KIs 7220 (48%) attended one of the 33 screening camps. The number of children attending each camp ranged from 119 to 369 with an average of 215 children.

### Prevalence of impairments and epilepsy

Of the 7220 screened, 2788 (39%) were identified as having at least one impairment/epilepsy as per the study case definitions (i.e. moderate/severe hearing or vision impairment, physical impairment, intellectual impairment, epilepsy). This gives a combined, estimated prevalence of impairments/epilepsy of 17.3/1000 children (95% CI: 16.9–17.7, Table 2). Extrapolating to the general population, suggests there are 9066 children per million population (all ages) with impairments/epilepsy in the study districts. These estimates are based on the assumption that the prevalence was the same for children who did and did not attend the screening camps. We also undertook a sensitivity analysis, assuming the prevalence among the 52% of children who did not attend the camps was 10% lower than those who did attend (15.0/1000), and then 10% higher (19.6/1000). The estimated prevalence of impairments/epilepsy was slightly higher in Thyolo: 19.8/1000 children (19.2–20.2) than Ntcheu: 15.5/1000 (14.9–16.1).

**Table 2:** Adjusted prevalence estimates of impairments/epilepsy in children in study area

Impairment/health condition	Number^a^	Prevalence per 1000 (95% CI)^a^	Prevalence per 1000 Range^b^	No. Per million total population^c^
Physical impairment	2247	6.6 (6.3–6.9)	5.8–7.6	3520
Hearing impairment ^d^	1550	4.6 (4.4–5.8)	4.0–5.3	2453
Visual impairment	243	0.7 (0.6–0.8)	0.7–0.8	373
Intellectual impairment	1452	4.3 (3.8–4.2)	3.7–4.8	2133
Epilepsy	1258	3.7 (3.5–3.9)	3.3–4.3	1973
Multiple impairments	806	2.4 (2.2–2.6)	2.1–2.7	1280
Any impairment/epilepsy	5844	17.3 (16.9–17.7)	15.0–19.6	9066

^a^The number and prevalence estimates are adjusted based on the assumption that the prevalence of disability was the same among children who did and didn’t attend the examination camp. The denominator used to calculate the prevalence of impairments is the total number of children (338,235) living in the 8 traditional areas included in the study

^b^The prevalence range is based on sensitivity analysis assuming the proportion of non-attenders having an impairment/epilepsy was ±10% of the actual proportion among attenders

^c^Per million total population of all ages, not population of children

^d^It was not possible to conduct Pure Tone Audiometry on 40% of children aged 5+ years and Ottoacoustic Emmission test results were used for those children

Among the 2788 children with an impairment/epilepsy, 48% were female and approximately half came from each district (53% from Thyolo, 46% from Ntcheu). The vast majority (93%) of children were from families with a monthly income of less than $30. Forty percent of all primary caregivers were illiterate and just under half (46%) had attended primary school. Only 7% of primary caregivers had attended secondary school. There was no significant difference in these variables between the two study districts.

### Impairment types

Based on the preliminary screening questions a total of 2475 children were screened for hearing impairment, 1082 for vision impairment, 1052 for intellectual impairment 1282, for physical impairment and 1165 for epilepsy.

Physical impairment was the commonest impairment type (39%) observed followed by bilateral hearing impairment (27%), intellectual impairment (26%), epilepsy (22%) and bilateral vision impairment (4%). Fifteen percent of the children had multiple impairments. The estimated prevalence, range and number per million population for each of the different impairments/epilepsy is shown in Table 2. The distribution of impairment types was broadly similar between the two districts.

### Physical impairment: Diagnosis and service needs

A total of 1265 diagnoses of physical impairment were made for 1094 children (some children had multiple diagnosis, Table 3). A neurological diagnosis was the most common (*n* = 591, 54%), followed by congenital e.g. club foot (*n* = 215, 20%), acquired non-traumatic e.g. angular limb deformity (*n* = 194, 18%) and trauma (*n* = 145, 13%) diagnoses. The most common health condition was cerebral palsy (accounting for a quarter of all children with physical impairment).

**Table 3:** Diagnosis of moderate/severe physical impairment

DIAGNOSIS	*N*	**%** ^a^
Polydactyly	13	1%
Syndactyly	10	1%
Other upper limb deformity	26	2%
Club foot	52	4%
Other lower limb deformity	58	5%
Upper and Lower Limb deformity	9	1%
Spine deformity	21	2%
Cleft lip or cleft palate	9	1%
Other congenital deformity	21	2%
Cause not given	5	0.4%
**TOTAL Congenital**	**224**	**18%**
Burn contracture	45	4%
Fracture malunion	15	1%
Head injury	1	0.1%
Recurrent/chronic dislocation	5	0.4%
Post traumatic joint stiffness	28	2%
Tendon/Muscle problem	4	0.4%
Peripheral nerve problem	15	1%
Amputation	16	1%
Cause not given	20	2%
**TOTAL Trauma**	**149**	**12%**
Epilepsy	56	4%
Developmental delay	59	5%
Cerebral Palsy	282	23%
Para/quadra/tetri/hemi-plegia	138	11%
Peripheral nerve palsy	25	2%
Other neurological	137	11%
Cause not given	2	0.2%
**TOTAL Neurological**	**698**	**55%**
Joint infection	5	0.4%
Bone infection	19	1.5%
Skin wound/infection	4	0.3%
TB spine/spine infection	1	0.1%
Degenerative joint infection	4	0.3%
Non infective non traumatic joint infection	1	0.1%
Bow legs	26	2%
Knock knees	38	3%
Other joint deformity	9	1%
Bone tumour	8	1%
Soft tissue tumour	13	1%
Skin tumour	5	0.4%
Spinal deformity – kyphosis	10	1%
Limb pain limiting function	8	1%
Other	39	3%
Cause not given	1	0.1%
**TOTAL Acquired non-traumatic**	**194**	**15%**

^a^Some children had multiple diagnoses. Percentages in this table are calculated out of the total number of diagnoses (*n* = 1265) rather than individual children

The most common services recommended following examination at the screening camp were physical therapy (44% of children with physical impairment) surgery (17%), occupational therapy (14%), wheelchair (8%), medication (7%) and appliances/orthosis (3%, data not shown). Extrapolating these data suggests that there are approximately 1600 children per million population who could benefit from physical therapy, 700 from surgery, 500 from occupational therapy and 300 from a wheelchair, and 250 from medication. Caregivers were asked if their child had previously received any of these services. The majority (60%) had not received any services in the past, 17% had previously had physical therapy and 12% had received surgery.

### Hearing impairment: Diagnosis and service needs

Just under half of the children (45%) with hearing impairment had a perforated ear drum and 36% had evidence of discharge in the middle ear. Wax was evident in one third of the children. Inflammation, foreign bodies, retraction and red/bulging ears were less common (<5%). The majority (73%) of children with bilateral hearing impairment had one or more of symptoms indicating conductive hearing loss (sound unable to pass from outer to inner ear, usually because of a blockage). The remainder did not have any of these symptoms indicating sensorineural causes of hearing loss (caused by damage to hair cells inside the inner ear or auditory nerve damage). Extrapolating these findings, we estimate that, there are approximately 1800 children per million population with conductive causes of hearing impairment that could be treated or prevented through the provision of basic primary ear and hearing care services.

### Vision impairment: Diagnosis and service needs

**Fig. 1:**
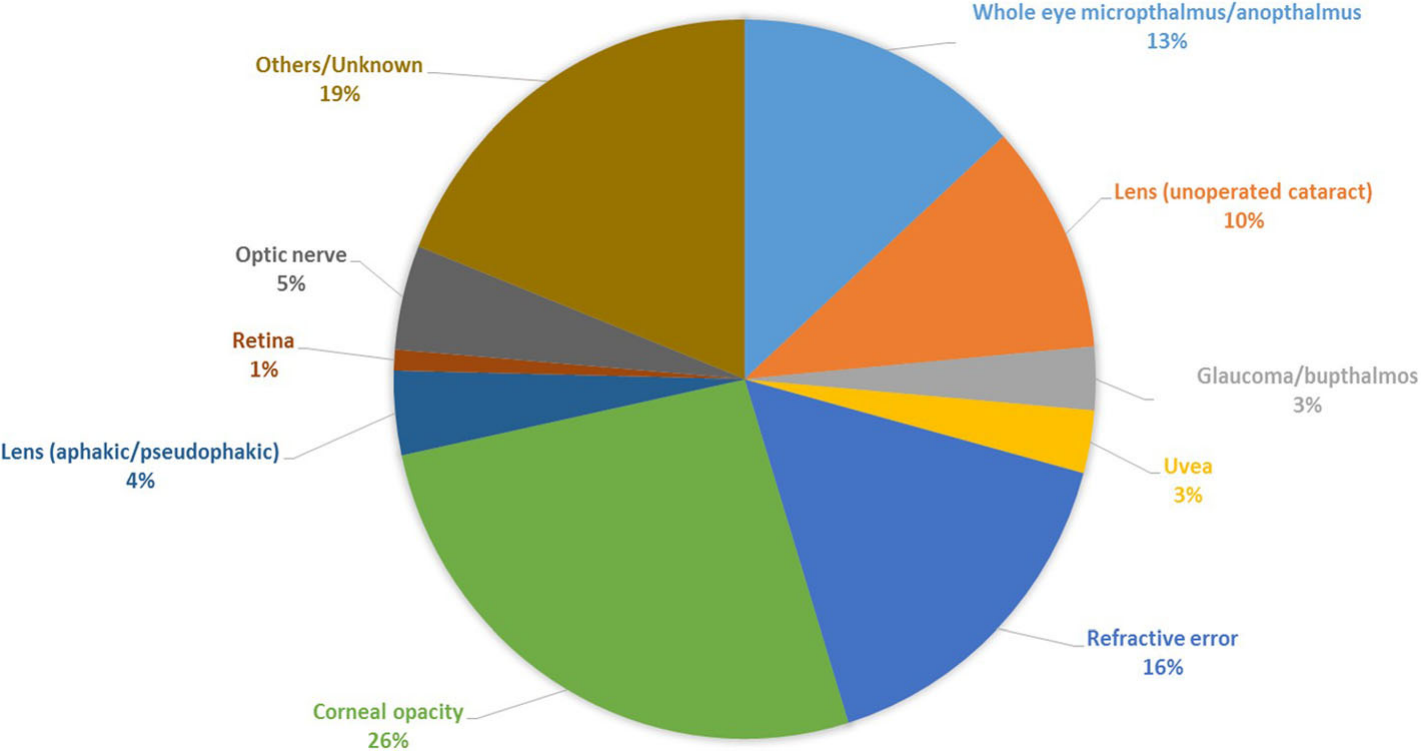
Causes of moderate/severe vision impairment

Corneal opacity was the leading known cause (Fig. 1), responsible for just over a quarter of moderate/severe bilateral vision impairment, followed by refractive error (16%), conditions of the whole eye (microphthalmus/anophthalmus) and un-operated cataract (10%). The proportion of childhood vision impairment due to corneal opacity was higher (32%) among children aged ≥10 years compared to <10 years (18%). In terms of service needs, extrapolating these data suggest that at least 60 children with moderate/severe vision impairment per million total population could benefit from refractive services and that 40 children per million total population need cataract surgery. Approximately 100 children per million population have corneal scars which could have been prevented through the provision of basic primary health services, prevention of Vitamin A deficiency and measles.

### Intellectual impairment

**Fig. 2:**
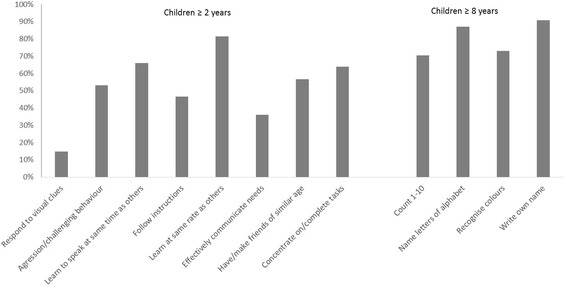
Proportion of children with intellectual impairment reported ‘unable’ to do each screening tool item

Of all the children identified as having intellectual impairment 15% were diagnosed as having Cerebral Palsy, 14% microcephaly, 9% hydrocephaly and 6% had Down’s Syndrome. Figure 2 shows the response distribution to the items in the intellectual impairment assessment tool for children identified as having an intellectual impairment.

### Epilepsy

Children identified as having epilepsy were asked about previous treatment sought. The majority of children with epilepsy (80%) were reported to have seen by a medical person but only half of the children were reported to be currently taking epilepsy medication.

### Multiple impairments

There were 424 children with multiple impairments of whom 87% were diagnosed with two impairments, 12% with three and 1% with four or five impairments. Just under a third of children with multiple impairments had cerebral palsy, a further 22% were diagnosed with intellectual impairments plus epilepsy, 21% with physical plus intellectual impairments, 9% with physical impairment plus epilepsy and 6% with hearing impairments plus intellectual impairments. Overall, 79% of children with multiple impairments had intellectual impairments, 69% had physical impairments, 46% had epilepsy, 15% had hearing 5% had vision impairments.

### Type of impairment and school attendance

Nearly three-quarters of children with an impairment/epilepsy that were of school-going age (age > 5 years) were attending school (73%). School attendance decreased with age (5–9 years: 77%, 10–14 years: 80%, 15–18 years: 57%). Among children attending school, 7% were at nursery school, 92% were at primary and 2% were at secondary school. Most of these children (82%) were in the correct school level for their age (e.g. 6–13 years for primary school and 14–17 years for secondary) although 15% of children aged more than 14 years were still attending primary school. Nearly all children (99%) were in mainstream education.

For children aged ≥5 years not attending school, the most common reason given was having an ‘illness’ lasting more than 1 month (44%, Table 4). Environmental (school not accessible) and attitudinal barriers (refused by school/negative attitudes of students) related to disability were reported for 17% of children.

**Table 4:** Reported reasons children of school-going age not currently attending school

Reason	*N*	%
Not enough money	11	3%
Lack of interest to go to school	32	8%
Illness lasting less than one month	72	18%
Illness last more than one month	174	44%
Because of disability: refused by school	28	7%
Because of disability: negative attitudes of students	16	4%
School not accessible	24	6%
Other	43	11%

*NB*: Multiple responses were allowed hence summed totals equal more than 100%

There was a significant difference in the proportion of children attending school between the types of impairment. Compared to children with hearing impairments (9% non-attendance) non-attendance was significantly higher for children with other impairments/epilepsy (physical impairment: odds ratio (OR) 2.0 95% Confidence Intervals (CI) 1.3–3.0; vision impairment: OR 2.3 95% CI 1.1–4.5; Epilepsy: OR 4.0 95%CI 2.6–6.1; intellectual impairment: OR 5.0 95%CI 3.3–7.6 and multiple impairments: OR 16.2 95% CI 10.7–24.5). This difference remained significant with adjustment for age, caregiver education and district (data not shown).

### Sub-study of non-attenders at the screening camp

A total of 212 households with 236 children listed by KIs but who had not attended a screening camp were interviewed to explore their reasons (response rate 80%). The age and sex distribution of this sub-sample was very similar to that of children with confirmed impairments/epilepsy who attended the camps (data not shown). Using the definition of ‘some problem with at least two domains or a lot of problem /cannot do with at least one domain’ [21] 63% of children were classified as having a disability (data not shown).

As shown in Table 5, nearly a third of respondents reported organisation/communication reasons for not attending including not knowing about the camps (31%), not knowing the time or date of the camp (15%) and attending the camp too late/forgetting (5%). Access difficulties were reported by over a third of respondents including distance to camp (17%), financial barriers (11%) and physical barriers (6%). Nearly half of respondents (44%) reported personal family reasons including being busy working, travelling, attending family/village events (19%), illness of child or another household member (16%) and no-one to take the child (6%).

**Table 5:** Reasons given for not attending screening camps

**Reason**	*N*	%
Organisation/communication reasons:		
Did not know about camp	65	31%
Did not know time/date of camp	32	15%
Attended camp too late / not examined	8	4%
Forgot time/location of camp	2	1%
Access difficulties:		
Camp too far	35	17%
No money – transport	18	8%
No money - incidentals (food en route, soap to wash clothes)	7	3%
Physical difficulties (e.g. child too heavy to carry, mother pregnant)	12	6%
No transport available	4	2%
Personal family reasons:		
Busy (working, away, attending family/village events)	40	19%
Child/household member ill.	33	16%
No one to take child	13	6%
Didn’t want child to miss school	6	3%
Other	10	5%

*NB*: Multiple responses were allowed hence summed totals equal more than 100%

## Discussion

This was the first large study to use the KIM to estimate the prevalence and causes of childhood impairment in Africa. This method successfully identified more than 2500 children with different types of impairments.

The prevalence for any impairment/epilepsy in this study was estimated to be 17.3/1000 children. This is higher than the KIM study in Bangladesh (conducted across three districts totalling approximately 600,000 people) which included children <18 years Bangladesh (9.0/1000) [10] and in Kenya (conducted in one district of approximately 100,000 people) which included children aged <10 years (7.5/1000) [23]. Both of these studies also involved community level KIs familiar with the local area who underwent one-day training covering the same topics. There are a number of possible reasons for the higher prevalence estimate in Malawi. Children with intellectual impairment were included in the current study, but not in Bangladesh. In the Kenya study the duration for finding children was shorter (2 weeks) and the examination was conducted in the child’s home by a paediatrician rather than by the range of clinicians included in Bangladesh and Malawi. Methodological issues with assessment of hearing impairment may also have contributed. Hearing impairment was acknowledged as a probable underestimate in Bangladesh where less than 20% of those with suspected hearing impairment could be assessed (using Pure Tone Audiometry or Otoacoustic Emissions Tests) [10]. In Kenya, hearing was assessed through questions and response to noise which may also underestimate hearing loss. In contrast, in the current study a slight over-estimation of moderate/severe hearing impairment cannot be ruled out. It was not possible to conduct Pure Tone Audiometry on 40% of children aged above 5 years and therefore we relied on Otoacoustic Emissions assessment for these children. Although this is considered a reliable screening tool [24], it does not measure level of hearing loss and therefore some children with mild hearing loss may have been included. Comparison with other multiple impairment studies is limited by the lack of available data and the different disability measurement used. Our estimates were lower than the 2008 Malawi Housing and population census (24/1000 children) which relied on self-reported disability [8] and a population based surveys in Cameroon (47/1000 children) and India (36/1000 children) which used both self-report functional limitations and clinical screening [25]. Using the Ten Question screening tool, the UNICEF Multiple Indicator Cluster Surveys conducted in 26 countries the found that 14–36% of children screened positive, considerably higher than our estimates [26]. However, this tool is acknowledged to have a relatively low positive predictive value, identifying children who with further examination are found not to have a clinically-detectable disabling impairment, and including children with less severe disabilities.

The prevalence estimates for the different impairment types were generally comparable to previous studies in LMIC, lending weight to the reliability of our findings. In line with previous research, physical impairment was the most common impairment type identified and cerebral palsy was the most common underlying health condition for children with physical impairment [3]. The prevalence of visual impairment was similar to estimates from previous studies using the KIM (for vision only) in Uganda (0.7/1000) and Ethiopia (0.6/1000) [27, 28]. The epilepsy prevalence in our study was within the range estimated in a systematic review of epilepsy in Sub-Saharan Africa. [29] The estimated prevalence of hearing impairment were lower than the estimates produced for the Global Burden of Diseases study for Sub-Saharan Africa (19/1000 for children aged 5–14 years), although as the authors acknowledge, these estimates are based on very limited population based data [30].

The data generated provide important information for service planning and advocacy for children with disabilities. We estimate that around 2100 children per million population in Malawi are in need of physical or occupational therapy, and that 300 children per million population could benefit from a wheelchair, but the data suggest a large unmet need for these services. A study assessing the capacity of hospitals to manage trauma and musculoskeletal impairment in the Eastern, Centre and Southern region, to which Malawi belongs, found that only a third of the district hospitals had rehabilitation services [31]. A country level situational analysis of availability of facilities, resources and personnel to meet these needs would be beneficial for informing planning of future service provision.

The study findings suggest that more than 75% of hearing impairment in children is attributable to conductive hearing loss caused by conditions such as middle ear infections and presence of wax. These conditions can easily be prevented and treated through primary ear and hearing care services. However, these services are currently limited in Malawi and there is an urgent need to increase the number of personnel trained in primary ear care in this setting. Potential innovative strategies to address this gap which could be explored include the training of primary level health workers, such as Health Surveillance Assistants, in the delivery of primary ear and hearing care.

Corneal opacities were the commonest cause of vision loss, as is typical in very low income settings [28]. Leading causes of corneal scars include Vitamin A deficiency and measles which can be prevented through provision of basic primary health care services and are therefore an urgent priority in Malawi. The proportion of visual impairment due to corneal scar was lower among the children aged <10 years (18%) compared to those ≥10 years (32%) suggesting some positive effect of the recent increase in measles immunization / vitamin A supplementation coverage in this setting. However, at 18% it was still high, indicating a need to strengthen and sustain these efforts. Refractive error was the second leading cause of vision impairment, highlighting a need for basic eye screening among children and provision of corrective glasses, which could be integrated into school health programmes [28].

This study found that only half of the children identified as having epilepsy reported receiving any medication, even though 80% had reportedly seen a health care professional for their condition. The significant treatment gap for epilepsy has been reported in other settings [32] Our study highlights the urgent need to explore and address the specific issues and barriers to treatment in this setting.

Intellectual impairment is a relatively neglected area in LMIC with services, trained personnel and evidence on the effectiveness of interventions all lacking [33]. There is some evidence to suggest that provision of psychosocial services by non-specialist providers (e.g. teachers and parents) may be effective for children with intellectual impairment where specialist services are unavailable [33]. This approach deserves exploration in Malawi given the relatively high number of children experiencing intellectual disability in this study. Furthermore, parent supported interventions such as for Cerebral Palsy can also fill an important gap [34].

The majority (73%) of children with disabilities in this study were attending school, which concurs with other studies [3]. Although encouraging, the fact that nearly 30% of children with disabilities of school-going age were not attending school, and the reasons for this, should not be ignored. Furthermore, information about the quality of education received was not collected. Environmental and attitudinal barriers were among the reported reasons for non-attendance. These need to be addressed through policy and school-level changes in order to achieve the inclusion of children with disabilities in education which is a fundamental right and so important for their future social and economic well-being.

The KIM method has been shown to be valid and cost-effective method for identifying children with impairments and generating important epidemiological information to inform service planning. A KIM working guide has been produced to facilitate individuals or organisations who wish to implement this method in other settings [35]. This working guide provides practical information on the different steps involved as well as the resources and personnel required.

### Limitations

Nearly 50% of children did not attend the screening camps and we therefore relied on assumptions (of the same impairment prevalence in this group as those who did attend) to generate a total prevalence estimate. This is supported to some extent by the sub-study that showed that 63% were classified as having a disability according to reported functioning. Reasons given for low attendance included organisational issues, physical and financial barriers and personal factors and these should be taken into consideration in future studies using this methodology.

A substantial proportion of children (61%) listed by the KIs did not subsequently screen positive for impairment/epilepsy suggesting a relatively low study specificity. This pattern was also observed in the KIM in Bangladesh [9]. There are a range of potential reasons for this. Our study had a relatively narrow focus on moderate and severe impairments and epilepsy and some of the children listed by KIs may have had mild impairments or unilateral vision/hearing impairments or other health conditions temporarily affecting body function/structures. It is also possible that given limited access to health care services among this population, children were referred by KIs to the camps because it was an opportunity to see a health professional for another health problem/acute condition. While preferable to under-referring of children with disabilities, the over-referral of children in this method does have time, resource and efficiency implication. We did not record socio-demographic data on the KIs (e.g. age, sex, socio-economic status) and this should be collected in future KIM studies.

The study focussed on clinical measures of impairment and epilepsy. This approach generates important information for planning specific treatment and rehabilitation services. However, it is acknowledged that it provides only part of the picture of disability because it does not capture an individual’s functioning and participation which can vary substantially depending on a range of internal and external factors [1]. We did not collect data on mental disorders in this study due to lack of available context appropriate tools and the questions used to assess intellectual impairment had not been previously validated. Since this study, a tool has been developed for assessing self-reported functioning in children: UNICEF/Washington Group Extended Set on Child Functioning [21, 25] which includes domains on anxiety and depression and intellectual functioning. Future applications of KIM could consider using this tool for comparability with other studies and to assess reported functioning in all domains including anxiety and depression and intellectual functioning.

### Strengths

This study identified a large number of children with impairments in Malawi and makes an important contribution to the limited data available on the epidemiology of child disability in LMIC. Intellectual impairment was included in this study, which has been lacking from previous KIM projects for child disability and for which data in Africa are generally scarce. The data on prevalence and aetiology for the individual impairment groups and epilepsy were comparable with the few previous studies that have been undertaken in LMIC. Community involvement is an important strength of this study methodology and five hundred community KIs underwent training which included disability awareness.

## Conclusions

This was the first study to use the KIM to estimate the prevalence of childhood disability in Africa. As well as providing epidemiological data on impairment in children and service needs that can be used to inform planning and advocacy of interventions for improving the quality of life of children with disabilities, this method study also has an important capacity building and disability awareness raising component.

## Abbreviations

CI: Confidence Interval
CRT: Convention on the Rights of the Child
ENT: Ear Nose and Throat
ICF: World Health Organisation International Classification of Functioning, Disability and Health
KIM: Key Informant Method
KIs: Key Informants
LMIC: Low and Middle Income Countries
OAE: Otoacoustic Emissions
OR: Odds Ratio
PTA: Pure Tone Audiometry
UNCRPD: United Nations Convention on the Rights of Persons with Disabilities
VA: Visual Acuity
