# Correction to: Childhood disability in Malawi: a population based assessment using the key informant method

**DOI:** 10.1186/s12887-018-1073-3

**Published:** 2018-02-28

**Authors:** Myroslava Tataryn, Sarah Polack, Linda Chokotho, Wakisa Mulwafu, Petros Kayange, Lena Morgon Banks, Christiane Noe, Chris Lavy, Hannah Kuper

**Affiliations:** 1 International Centre for Evidence in Disability, London School of Hygiene & Tropical Medicine, WC1E 7HT, London, UK; 2 Beit Cure International Hospital, Blantyre, Malawi; 3 Department of Surgery, College of Medicine, University of Malawi, Blantyre, Malawi; 4 Department of Surgery, Opthalmology Unit, College of Medicine, University of Malawi, Blantyre, Malawi; 5 CBM, Bensheim, Germany; 6 Nuffield Department of Orthopaedics Rheumatology and Musculoskeletal Science, Oxford University, Oxford, UK

## Correction

Following the publication of this article [1] it was brought to our attention that inadvertently the COSECSA Oxford Orthopaedic Link (COOL) programme was not acknowledged for funding this study.

The completed ‘Acknowledgements’ section of the article should, therefore, read: “We are very grateful to the following organisations who provided the funding for this study: COSECSA Oxford Orthopaedic Link (COOL) programme funded by the UK Department for International Development, Cure International UK, Fight for Sight and Liliane Foundation. Representatives from these organisations were part of the advisory committee for the study, which gave guidance towards conduct of the study but were not involved the data collection, data analysis, interpretation of results, preparing the manuscript or the decision to publish. One of the authors (CN) works for CBM (who cofunded the study) and contributed to the conception of the study, interpretation of findings and reviewing the manuscript. No other funding bodies played a role in the design analysis, interpretation or writing of the manuscript or the decision to submit the manuscript for publication. We would also like to thank Professor Clare Gilbert and Professor Andrew Smith from the London School of Hygiene & Tropical Medicine for their input on the vision and hearing data collection and interpretation of results”.
